# Helicobacter pylori and Epstein-Barr Virus Coinfection Stimulates Aggressiveness in Gastric Cancer through the Regulation of Gankyrin

**DOI:** 10.1128/mSphere.00751-21

**Published:** 2021-09-29

**Authors:** Dharmendra Kashyap, Budhadev Baral, Shweta Jakhmola, Anil Kumar Singh, Hem Chandra Jha

**Affiliations:** a Department of Biosciences and Biomedical Engineering, Indian Institute of Technology Indore, Indore, India; b Biological Science and Technology Division, CSIR-Northeast Institute of Science and Technology, Jorhat, India; University of North Carolina, Chapel Hill

**Keywords:** *Helicobacter pylori*, Epstein-Barr virus, gankyrin, gastric cancer, coinfection

## Abstract

Persistent coinfection with Helicobacter pylori and Epstein-Barr virus (EBV) promotes aggressive gastric carcinoma (GC). The molecular mechanisms underlying the aggressiveness in H. pylori and EBV-mediated GC are not well characterized. We investigated the molecular mechanism involved in H. pylori- and EBV-driven proliferation of gastric epithelial cells. Results showed that the coinfection is significantly more advantageous to the pathogens as coinfection creates a microenvironment favorable to higher pathogen-associated gene expression. The EBV latent genes *ebna1* and *ebna3c* are highly expressed in the coinfection compared to lone EBV infection at 12 and 24 h. The H. pylori-associated genes 16S rRNA, *cagA*, and *babA* were also highly expressed during coinfection compared to H. pylori alone. In addition, upregulation of gankyrin, which is a small oncoprotein, modulates various cell signaling pathways, leading to oncogenesis. Notably, the knockdown of gankyrin decreased the cancer properties of gastric epithelial cells. Gankyrin showed a similar expression pattern as that of *ebna3c* at both transcript and protein levels, suggesting a possible correlation. Further, EBV and H. pylori created a microenvironment that induced cell transformation and oncogenesis through dysregulation of the cell cycle regulatory (*ccnd1*, *dapk3*, *pcna*, and *akt*), GC marker (*abl1*, *tff-2*, and *cdx2*), cell migration (*mmp3* and *mmp7*), DNA response (*pRB*, *pten*, and *p53*), and antiapoptotic (*bcl2*) genes in infected gastric epithelial cells through gankyrin. Our study provides a new insight into the interplay of two oncogenic agents (H. pylori and EBV) that leads to an enhanced carcinogenic activity in gastric epithelial cells through overexpression of gankyrin.

**IMPORTANCE** In the present study, we evaluated the synergistic effects of EBV and H. pylori infection on gastric epithelial cells in various coinfection models. These coinfection models were among the first to depict the exposures of gastric epithelial cells to EBV followed by H. pylori; however, coinfection models exist that narrated the scenario upon exposure to H. pylori followed by that to EBV. We determined that a coinfection by EBV and H. pylori enhanced the expression of oncogenic protein gankyrin. The interplay between EBV and H. pylori promoted the oncogenic properties of AGS cells like elevated focus formation, cell migration, and cell proliferation through gankyrin. EBV and H. pylori mediated an enhanced expression of gankyrin, which further dysregulated cancer-associated genes such as cell migratory, tumor suppressor, DNA damage response, and proapoptotic genes.

## INTRODUCTION

Gastric cancer (GC) is the world’s fourth leading cause of cancer-related deaths in both males and females (1). Helicobacter pylori and Epstein-Barr virus (EBV) are group 1 carcinogens that potentially contribute to the development of GC (2). While doing so, the microorganisms could alter gastric physiology and immunology (3). H. pylori is prevalent in about half of the global population, but it is known to cause GC in only 3% of the infected individuals (4, 5). The discrepancy in H. pylori infection and GC might be due to its strain variabilities such as differential expression of *cagA*, *vacA*, and *babA* (6–8). In addition, EBV is the second most prominent cancer-associated pathogen, involved in several types of lymphoid and epithelial cancers (9). Latent (*ebna1*, *ebna2*, *ebna3c*, *lmp1*, *lmp2a*, and *lmp2b*) and lytic (*bzlf1* and *gp350*) gene expression associated with EBV can alter the homeostasis of host gene expression (9). The viral protein also dysregulates cellular processes like cell cycle regulation, inflammation, angiogenesis, and hypermethylation (10, 11). Earlier studies have shown the ability of EBV to cause oncogenic transformation of primary gastric epithelial cells (12, 13). Thus, it is considered not only a passive carrier but also an active oncogenic virus contributing to early events in the development of GC (2). Additionally, it is known that lone infection by H. pylori or EBV is less severe in comparison to coinfection and takes more time to initiate GC development (2).

**FIG 1:**
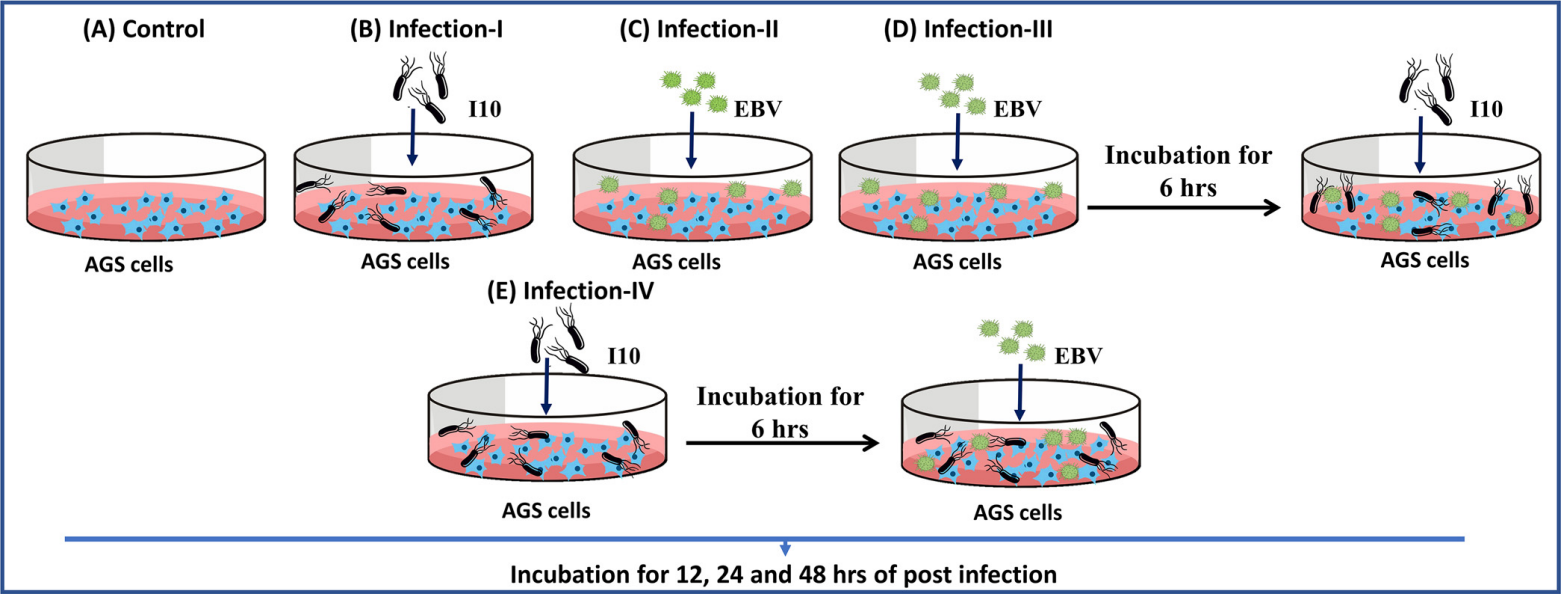
Coinfection models of H. pylori and EBV depict insight into the pathophysiology of gastric cancer. Illustration of all the studied infection models. AGS cells were cultured in 6-well plates, and I10 and EBV were directly incubated with these cells. (A) Uninfected AGS control cells. (B) Infection-I portrayed the infection by I10. (C) Infection-II AGS cells were infected with EBV only. (D) Infection-III AGS cells were infected sequentially, first exposing the cells to EBV for 6 h and then incubating the exposed cells with I10. (E) Infection-IV AGS cells were first exposed to I10 and then incubated with EBV. All the infected cells were further incubated for 12, 24, and 48 h. These infection models were used for the whole of this study except colony formation assay.

Moreover, in various cancers, gankyrin or PSMD-10, a recently discovered small oncogenic protein of 25 kDa, is known to be overexpressed (14). It is involved in various cellular processes such as cell cycle regulation, apoptosis, and tumorigenesis (15). Gankyrin negatively regulates protein retinoblastoma (pRb) and tumor suppressor protein-53 (p53/TP53) to execute its oncogenic functions (16, 17). Furthermore, a study reported that knockdown of gankyrin in GC increased the cell chemosensitivity to 5-fluorouracil and cisplatin by regulating cell cycle-related protein (18). Nonetheless, there is a paucity of studies which describe the relationship between gankyrin and pathogens, i.e., H. pylori and EBV. Thus, we evaluated the correlation between H. pylori- and EBV-associated pathogenic factors and the gankyrin oncogene. Furthermore, we determined an insightful relationship within the microbial niche which could modulate host gene expression, thus influencing the host pathophysiology. We determined a molecular mechanism to elucidate the unexplored strategy, which involved the participation of H. pylori in EBV-driven proliferation of gastric epithelial cells. In the present study, we used the AGS human gastric epithelial cell line, which is an excellent system with a potential to mimic the human gastric epithelium (except pH [1.5 to 3.5]). For the understanding of interplay between H. pylori and EBV, we developed four different infection schemes including uninfected controls. In the current study we used H. pylori strain I10. Briefly, the infection models are as follows: we took uninfected AGS cells as controls (Fig. 1A), while the infection schemes I and II (infection-I and -II, respectively) represent the exposure of AGS cells to I10 and EBV, respectively (Fig. 1B and C). Importantly, model III represented the sequential infection, i.e., AGS cells first exposed to I10 for 6 h and then to EBV (Fig. 1D); conversely, in model IV first we exposed the AGS cells to EBV for 6 h and then exposed them to I10 (Fig. 1E). We elucidated that EBV and H. pylori coinfection increased gankyrin expression, which possibly led to the proliferation of infected cells in the abovementioned scenarios. Furthermore, we determined the status of cell migratory, cell cycle regulatory, DNA repair, apoptosis, and tumor suppressor genes in coinfection models, which can be directly linked to the aggressiveness of carcinogenic properties.

## RESULTS

### Coinfection by H. pylori and EBV altered pathogenic gene expression in gastric epithelial cells.

It has been reported that I10 and EBV take 4 to 6 h to infect the epithelial cells (19, 20). Prior to the incubation of I10 with AGS cells, we performed Gram staining and observed a regular spiral shape of the bacteria (see Fig. S1 at https://drive.google.com/file/d/1co1IRzbSxg8RTYYbUVCuJN1_xxdnX92t/view?usp=sharing). Our results showed that infection-III and -IV produced a significantly favorable microenvironment for the propagation of the pathogens compared to the infection by a single pathogen. Coinfection also favored higher pathogen-associated gene expression than did the individual infection at a time. The expression of EBV-associated carcinogenic genes was multifold higher in coinfected cells than in the cells exposed to individual infection by either EBV or I10. Thus, we could conclude that both EBV and I10 synergistically provided a suitable milieu for the growth of each other. Subsequently, we also studied all the four infection conditions to understand the influence and severity of I10 and EBV in GC progression. Meanwhile, postcoinfection, we collected the cells at various time points (12, 24, and 48 h) and checked the profiles of the few important pathogenic and host genes (Fig. 1A, B, C, D, and E).

**FIG 2:**
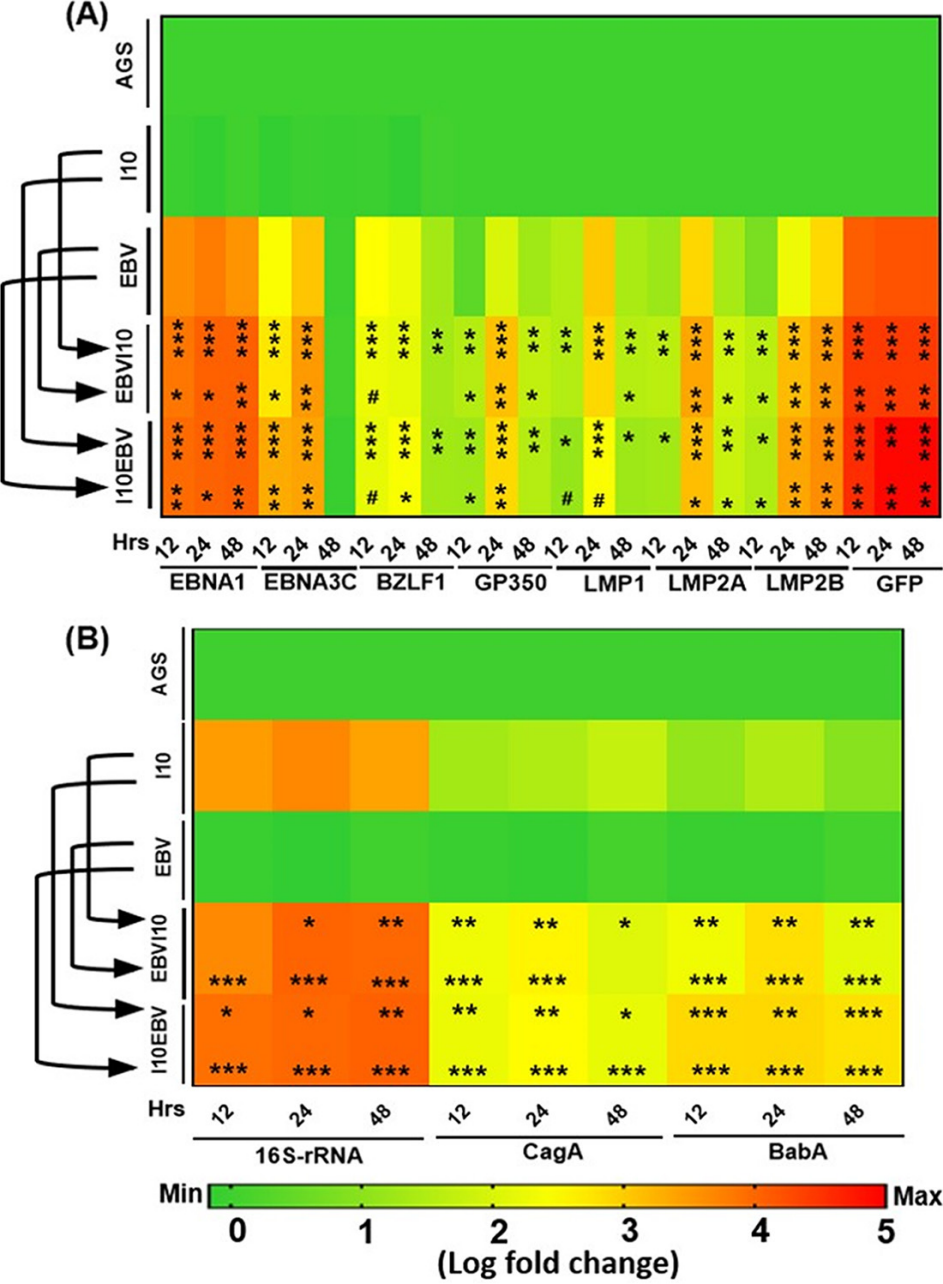
Interplay between H. pylori and EBV synergistically increases the transcript of their associated pathogenic genes. Heat map represents the log fold change of relative transcript expression of EBV-associated latent (*ebna1*, *ebna3c*, *lmp1*, *lmp2a*, and *lmp2b*) and lytic (*bzlf1* and *gp350*) genes and EBV-tagged *gfp* gene (A). Furthermore, the relative transcripts of I10-associated signature 16S rRNA, pathogenic cytotoxin-associated gene A (*cagA*), and blood group antigen binding adhesin A (*babA*) in all the infection models for 12-, 24-, and 48-h postinfection samples are shown (B). The experiment was performed for two biological and two technical replicates, and the results are shown as the mean ± SD for two independent experiments.

After collection of I10- and EBV-treated samples, we performed qRT-PCR of EBV latent (*ebna1*, *ebna3c*, *lmp1*, *lmp2a*, and *lmp2b*) and lytic (*gp350* and *bzlf1*) genes. Our results showed that *ebna1* transcripts were elevated in infection-III and -IV at 12 and 48 h compared to infection-I and -II (Fig. 2A). Similarly, the expression of *ebna3c* was higher in infection-IV at 12 h than in infection-I and -II. In the case of 24-h coinfected samples, the expression of *ebna3c* was also higher in infection-III and -IV than in infection-II and -I, showing the possible interplay between I10 and EBV. The expression of *ebna3c* significantly dropped at 48 h postinfection in infection schemes II, III, and IV in comparison to 12- and 24-h-postinfection samples (Fig. 2A).

Furthermore, the expression of *bzlf1* was high in infection-II, while in the case of infection-III and -IV strategies at 12 h postcoinfection the expression was reduced. A similar expression of *bzlf1* was observed in infection-II and -III at 24 h postinfection (hpi) compared to infection-IV. After 48 h of coinfection, a slightly higher expression of *bzlf1* was noticed in infection-II relative to infection-III and -IV (Fig. 2A). Moreover, we determined a higher expression of an EBV lytic gene (*gp350*) in the cells upon exposure to infection-III and -IV at 12 and 24 hpi in comparison to infection-I and -II. Also, the expression of *gp350* was higher at 12 and 24 h postinfection in infection-III and -IV compared to infection-I and -II, although the levels were alleviated at 48 h compared to infection-II (Fig. 2A). Additionally, we checked the expression of latent membrane protein genes such as *lmp1*, *lmp2a*, and *lmp2b* in the infection models III and IV and observed that the expression of these latent genes was significantly higher at 12, 24, and 48 h in comparison to the infection-I strategy (Fig. 2A). The expression of *lmp1* was slightly higher at 12 and 48 h in the cells in infection-III compared to infection-II (Fig. 2A). Meanwhile, the expression of *lmp1* was significantly downregulated at 24 h in infection-IV compared to infection-II (Fig. 2A). Also, we determined the expression of *lmp2a* and *lmp2b* was significantly higher at 24 and 48 h in infection-IV in comparison to infection-II. Furthermore, to understand whether the coinfection milieu also supported EBV DNA replication, we analyzed the expression of *gfp*. Interestingly, we observed an elevated expression of *gfp* transcripts in infection-III and -IV in comparison to infection-I and -II models of infection in all the studied time intervals (Fig. 2A). Moreover, the coinfection conditions somehow created favorable conditions for an increased expression of EBV-associated carcinogenic genes and elevated the EBV copy numbers signified by an increased expression of *gfp*.

Additionally, we investigated the expression of transcripts of I10-associated signature genes such as 16S rRNA, *cagA*, and *babA*. Our findings showed an elevated expression of 16S rRNA transcripts in coinfected samples compared to that of lone I10 infection (infection-I). Strikingly, in infection-III we observed similar expression of 16S rRNA as in infection-I at 12 hpi, while the expression of 16S rRNA transcripts was higher in infection-IV at 12 hpi. Moreover, the expression of 16S rRNA transcripts was almost double at 24 and 48 h compared to 12 hpi in infection-III and -IV. Besides, the expression of 16S rRNA transcripts increased successively from 12 to 48 h (Fig. 2B). Following qRT-PCR, we determined that the expression of *cagA* transcripts was significantly elevated at 12, 24, and 48 h in infection-III and -IV in comparison to infection-I and -II (Fig. 2B). Surprisingly, we also observed an elevated expression of *babA* at 12, 24, and 48 h in infection-I, while it significantly decreased in infection-II, -III, and -IV at 12, 24, and 48 h, respectively (Fig. 2B).

### EBV and H. pylori coinfection regulated the expression of gankyrin.

**FIG 3:**
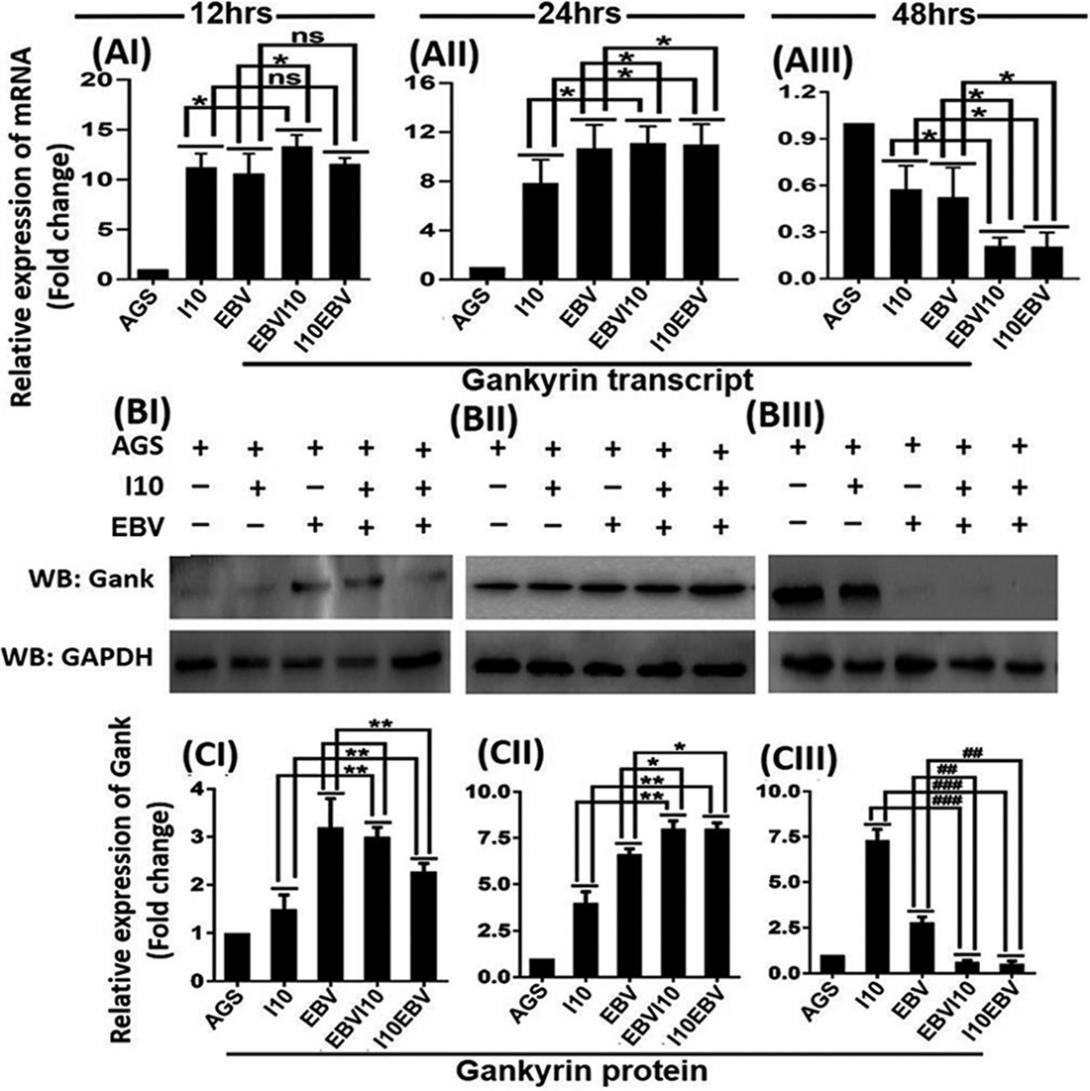
Coinfection of H. pylori and EBV upregulates the oncogenic protein gankyrin at both transcript and protein levels. Relative transcript expression of gankyrin in coinfection models depicts the expression of gankyrin at 12, 24, and 48 h (AI, AII, and AIII, respectively). Western blot image of protein gankyrin for 12-, 24-, and 48-h postinfection samples (BI, BII, and BIII, respectively). Further, the quantitative representation of Western blot image by Image J software and representative graph presented in terms of fold changes for 12-, 24-, and 48-h postinfection samples (CI, CII, and CIII, respectively). Interestingly, significantly higher expression of gankyrin was observed in 12- and 24-h postinfection samples, while the expression of gankyrin was significantly downregulated in infection-II, -III, and -IV in 48-h postinfection samples. The experiment was performed for two biological and one technical replicate, and the results are shown as the mean ± SD from two independent experiments. GAPDH, glyceraldehyde-3-phosphate dehydrogenase.

In addition to pathogen-associated factors, we also determined the host factors which could possibly be modulated by the microenvironment influenced by the presence of I10 and EBV. Principally, we investigated if gankyrin was associated with pathogen (I10 and EBV)-mediated GC. Interestingly, the infection by I10 and EBV mediated an enhanced expression of host-associated oncogenic gankyrin gene at transcript and protein levels. We determined slightly increased expression of gankyrin at 12 and 24 h in infection-III and -IV compared to infection-I and -II (Fig. 3AI and AII). At 48 hpi we observed a significantly lower expression of gankyrin in infection-III and -IV (Fig. 3AIII). Notably, we determined a similar pattern of protein gankyrin unlike infection-III and -IV at 12 hpi; the representative blot image is shown and quantified in Fig. 3BI, BII, and BIII and Fig. 3CI, CII, and CIII, respectively.

**FIG 4:**
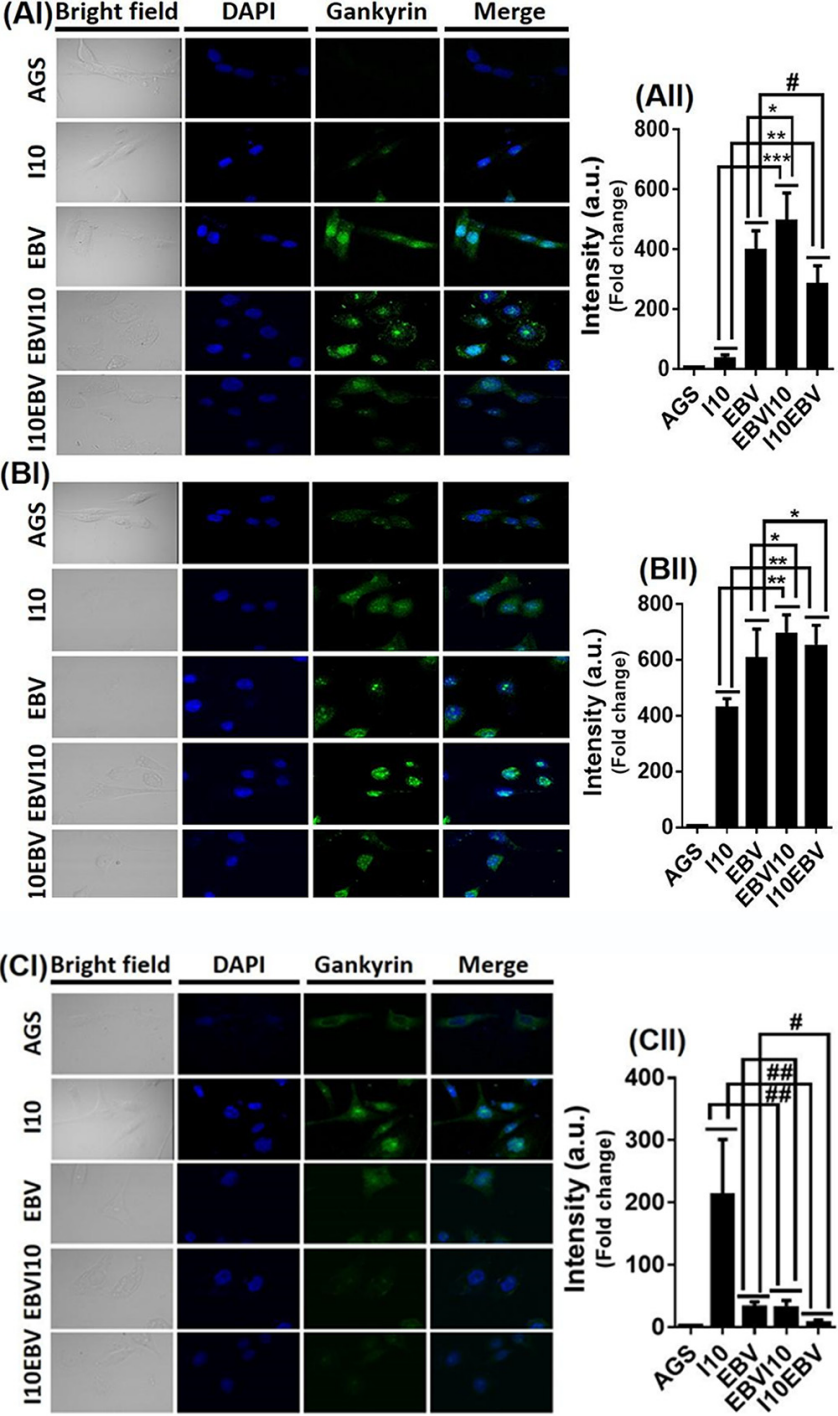
Coinfection by EBV and H. pylori causes gastric cancer through the upregulation of oncogenic protein gankyrin. Immunofluorescence results for the oncogenic gankyrin gene validate our previous transcripts and Western blotting results. Graphical representation of quantified immunoblot image through the Image J software. After the coinfection by I10 and EBV, we observed elevated protein expression of gankyrin at all the time points for all studied infections except 48 h postinfection, in which we observed downregulation of gankyrin in infection-II, -III, and -IV. (AI, BI, and CI) Representative immunoblot image of gankyrin represents the protein expression of gankyrin after 12, 24, and 48 h postinfection, respectively. First row shows an uninfected AGS cell. Second and third rows illustrate the gankyrin protein expression in infection-I and -II, while the fourth and fifth rows show the intensity of gankyrin expression after infection-III and -IV. (AII) Approximately 400- to 500-fold-increased expression of gankyrin observed in infection-II and -III after 12 h postinfection. (BII) The expression pattern of gankyrin in 24-h postinfection samples is about 600- to 650-fold higher in infection-III and -IV. (CII) The expression of oncogenic protein gankyrin after 48 h postinfection is about 200-fold higher in infection-I. Furthermore, the expression of gankyrin in infection-II is about equal to that in infection-III, while 25- to 30-fold-lower expression of gankyrin is observed in infection-IV. The experiment has been performed for two biological and three technical replicates, and the results are shown as the mean ± SD from two independent experiments.

Coinfection by I10 and EBV enhanced the expression of oncogenic protein gankyrin in a time-dependent manner. Here, we found that a coinfection by I10 and EBV may potentially favor the development and progression of GC through gankyrin. To support qRT-PCR and Western blotting results, which showed an upregulated expression of the gankyrin gene, we performed gankyrin immunostaining in the cells upon exposure to I10 and EBV according to the abovementioned infection strategies (infection-I, -II, -III, and -IV). A similar pattern of gankyrin expression was observed. A noticeable expression of gankyrin was observed in all four infection models. Comparably in infection-III and -IV, we recorded a significantly higher expression of gankyrin in 12-hpi samples (Fig. 4AI and AII). Also, in 24-hpi samples the expression of gankyrin remained higher in infection-III and -IV than in infection-I and -II (Fig. 4BI and BII). A significant alleviation in the expression of gankyrin in infection-III and -IV in 48-hpi samples in comparison to infection-I and -II was determined (Fig. 4CI and CII).

### EBV and H. pylori promoted the oncogenic properties of gastric epithelial cells by altering the expression of cell cycle regulators, tumor suppressors, gastric cancer markers, and cell migratory and apoptotic genes.

**FIG 5:**
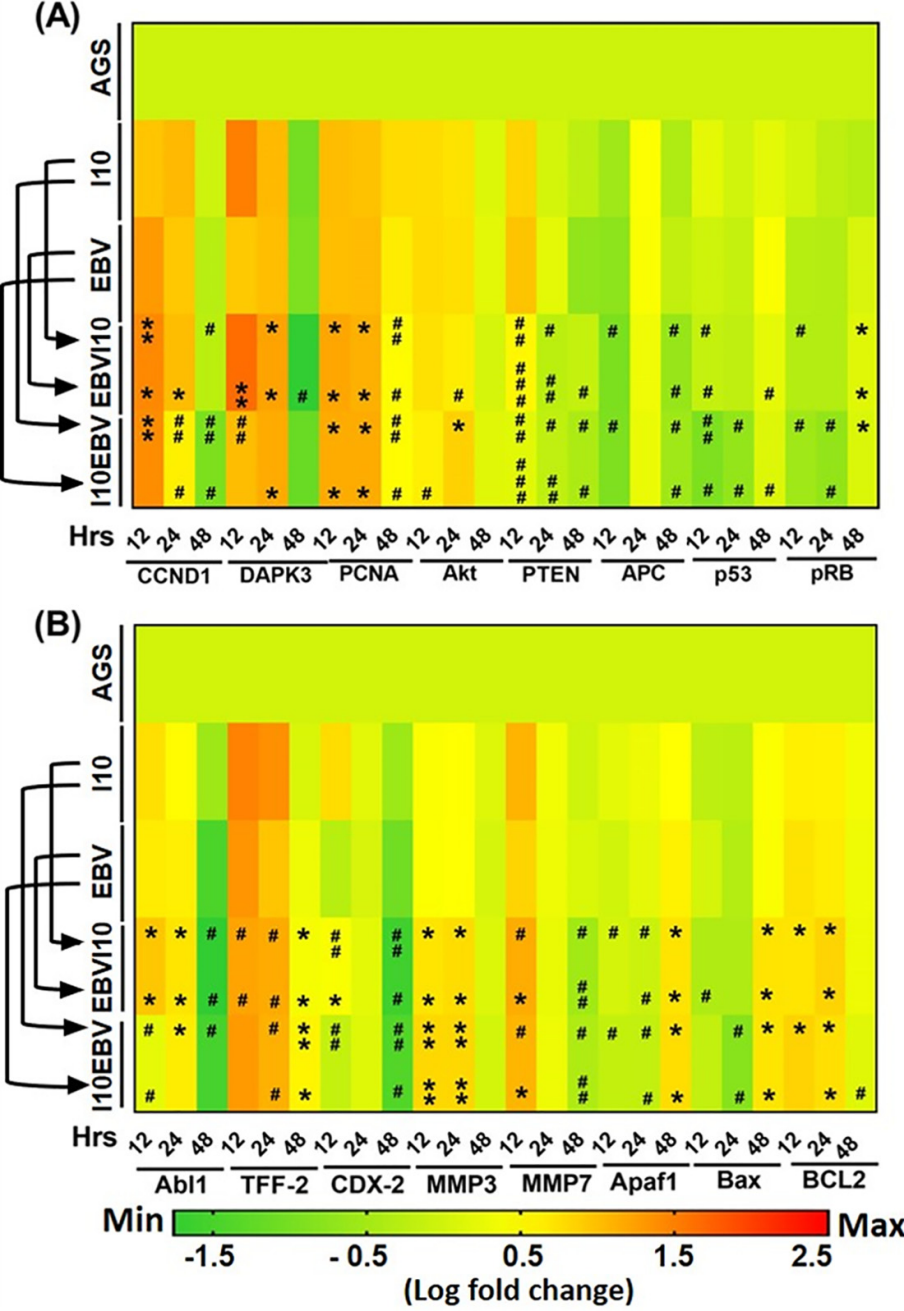
Coinfection by H. pylori and EBV promotes aggressiveness of gastric epithelial cells by the modulation of transcript expression of various cell signaling genes. (A) Heat map represents log fold change expression profiles of cell cycle regulators, *viz*., cyclin D1 (*ccnd1*), death-associated protein kinase 3 (*dapk3*), proliferating cell nuclear antigen (*pcna*), and AKR thymoma (*akt*). This heat map also illustrates the expression profiles of tumor suppressor phosphatase and tensin homolog (*pten*), adenomatous polyposis coli (*apc*), protein 53 (*p53*), and protein retinoblastoma (*pRB*) genes in all the four infection models including uninfected AGS cells at 12-, 24-, and 48-h time points. (B) Expression profiles of gastric cancer marker tyrosine protein kinase (*abl1*), trefoil factor-2 (*tff-2*), and transcription factor (*cdx-2*). Meanwhile, this heat map also represents the expression profiles of cell migratory matrix metalloprotease 3 (*mmp3*), matrix metalloprotease 7 (*mmp7*), and DNA damage response genes apoptotic protease activating factor 1 (*apaf1*), *bcl2*-associated x protein (*Bax*), and B-cell lymphoma 2 (*bcl2*). The experiment was performed for two biological and two technical replicates, and the results are shown as the mean ± SD from two independent experiments.

Through qRT-PCR we recorded an elevated expression of the cyclin D1 gene (*ccnd1*) in infection-III and -IV at 12 and 24 h compared to samples treated with individual pathogens (infection-I and -II) (Fig. 5A), while we witnessed a significantly lower expression of *ccnd1* at 48 h in infection-III and -IV compared to infection strategies I and II (Fig. 5A). Also, we observed an increased expression of the death-associated protein kinase 3 gene (*dapk3*) after 12 hpi in infection-I and -III compared to infection-II and -IV (Fig. 5A), while in the 24-hpi scenario we noticed a higher *DAPK3* expression in infection-III and -IV than in infection-I and -II (Fig. 5A). Nonetheless, an opposite expression pattern was observed at 48 hpi in comparison to 12 and 24 h (Fig. 5A). Notably, an elevated expression of the proliferating cell nuclear antigen gene (*pcna*) was observed at 12, 24, and 48 hpi compared to its uninfected AGS cell control, while a reduced expression of *pcna* was examined at 48 hpi in infection-III and -IV in comparison to infection-I and -II (Fig. 5A). Furthermore, the expression of *akt* was comparatively less in infection-III and -IV at 12 hpi.

Additionally, we determined the expression of tumor suppressor genes. We noted decreased expression of *pten* in infection-III and -IV in comparison to infection-I and -II at 12, 24, and 48 hpi (Fig. 5A). Additionally, we examined the expression of *apc*, another tumor suppressor gene, which performs its function upon interaction with cell adhesion molecules like E-cadherin. It is also known to negatively regulate the expression of beta-catenin. We observed a decreased expression of *apc* in 12-hpi samples from all four infection models (Fig. 5A). The expression of *apc* was elevated after 24 h post-pathogen incubation in infection models I, II, III, and IV (Fig. 5A). Furthermore, in 48-h infected samples, we determined a higher expression of *apc* in infection-II followed by infection-I and -IV (Fig. 5A). Also, we noticed a decreased expression of another well-known tumor suppressor gene, *p53*, in infection-III and -IV in comparison to infection-I and -II at 12 and 24 hpi (Fig. 5A), while in 48-hpi samples, *p53* transcripts were higher in infection-II than in infection-I, -III, and -IV (Fig. 5A). Furthermore, analysis of the *pRb* gene revealed alleviated expression in infection-II, -III, and -IV at 12 and 24 hpi, unlike infection-I (Fig. 5A). Meanwhile, an opposite expression pattern was observed at 48 hpi (Fig. 5A).

The expression of gastric cancer gene *abl1* was significantly higher in infection-III than in infection-I and -II at 12 hpi, while expression was decreased in infection-IV (Fig. 5B). Further, we determined an increased *abl1* expression in all infection models in comparison to uninfected control after 24 hpi (Fig. 5B). Surprisingly, the expression of *abl1* was downregulated in infection-III and -IV compared to infection-I and -II after 48 hpi (Fig. 5B).

A reduction in expression of *tff-2* was observed at 12 and 24 h in infection-III and -IV compared to infection-I and -II (Fig. 5B). Also, in 48-h-postinfection infection-III and -IV samples increased expression of *tff-2* was noticed (Fig. 5B). The expression of *cdx2* was alleviated in infection-III and -IV at all the time points compared to infection-I. Moreover, we noted a decreased expression of *cdx2* while comparing infection-III and -IV at 12, 24, and 48 h with infection-II (Fig. 5B).

The upregulation of cell migratory genes is a hallmark of cancer cell metastasis. Interestingly, we recorded a significantly elevated expression of *matrix metalloproteinase 3* (*mmp3*) in infection-III and -IV in comparison to infection-I and -II in 12- and 24-h infected samples (Fig. 5B). Additionally, after 48 hpi the expression of *mmp3* was moderately high in infection-I, while in infection-II, -III, and -IV, the expression of these transcripts was similar to that in uninfected control (Fig. 5B). The results revealed that expression of *matrix metalloprotein 7* (*mmp7*) was upregulated in infection-III and -IV in contrast to infection-II after 12 hpi. We noted a significantly reduced expression of *mmp7* at 48 hpi (Fig. 5B).

The function of DNA damage response genes in humans is to provide stability and maintain genome integrity. Moreover, cancer-causing infectious agents such as I10 and EBV can change the status of DNA damage response genes. We determined that the expression of DNA damage response gene *apaf1* was slightly lower in infection-III and -IV in 12- and 24-h infected samples (Fig. 5B). Surprisingly, we observed an elevated expression of *apaf1* at 48 h in the case of infection-III and -IV (Fig. 5B). We noticed an alleviated expression of *bax* at 12 and 24 h (Fig. 5B). *bax* was highly expressed in infection-III and -IV in comparison to infection-I and -II after 48 hpi (Fig. 5B). In addition to proapoptotic gene expression, the DNA damage response genes also execute their function by regulating the antiapoptotic properties. The upregulation of antiapoptotic genes such as *bcl2* helps in cell survival and proliferation. Pathogens such as I10 and EBV modulate the expression of these host proteins and promote the severity of various cancers including GC. Compared to the uninfected controls, the coinfected samples showed an upregulated expression of *bcl2* at 12 hpi in the infection models III and IV in comparison to uninfected control, whereas a higher expression of *bcl2* was observed in infection-IV (Fig. 5B). A similar expression of *bcl2* was observed in 24-h infected samples, unlike infection-III, in which we detected a higher expression of *bcl2* (Fig. 5B). It was observed that in 48-h-postinfection samples the expression of *bcl2* was still higher than in the uninfected control (Fig. 5B). These results revealed that the expression of *ebna3c* and the gankyrin gene might regulate various cell regulator and tumor suppressor genes. Endogenous and exogenous expression of gankyrin elevated the cancer properties of gastric epithelial cells.

**FIG 6:**
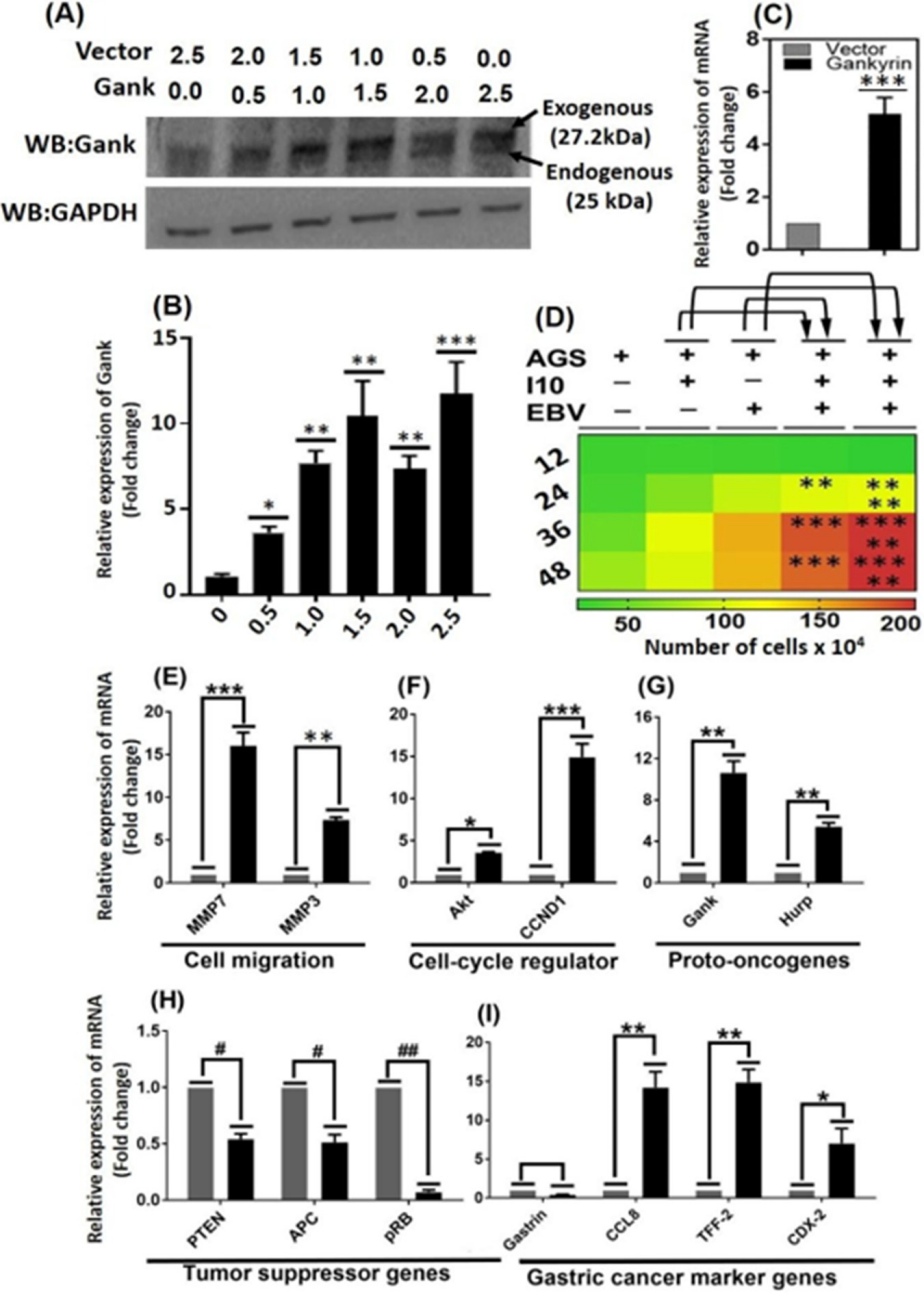
Coinfection-mediated and ectopic expression of gankyrin in AGS cells promotes the cell proliferation by interfering with the expression of various cell-signaling-associated cellular genes. (A) Representative Western blot showing the ectopic expression of oncogenic protein gankyrin. (B) Graphical representation of increased concentration gradient of gankyrin quantified by the Image J software of Western blot image. (C) Ectopic expression of gankyrin enhances the cell proliferation in trypan blue cell exclusion method of cell counting. (D) Heat map represents that infection by I10 and EBV also enhances the rate of cell proliferation in all studied infection models and may potentially be linked with the increased expression of gankyrin. (E) Ectopic expression of gankyrin significantly enhanced the expression of cell migratory genes *mmp7* and *mmp3*. (F and G) Representative graph shows the elevated expression of cell cycle regulatory genes *akt* and *ccnd1* and protooncogene hepatoma upregulated protein (*hurp*), respectively. (H) Moreover, overexpression of gankyrin significantly alleviates the expression of tumor suppressor genes *pten*, *apc*, and *pRB*. (I) Meanwhile, the expression of gastric cancer marker gastrin gene expression is slightly lower followed by the upregulation of C-C motif chemokine ligand-8 (*ccl-8*), *tff-2*, and *cdx-2*. The experiment was performed for two biological and two technical replicates, and the results are shown as the mean ± SD from two independent experiments.

Protein analysis through Western blotting showed an exogenously overexpressed gankyrin in AGS cells after 48 h after transfection of vector containing gankyrin (Fig. 6A and B and see Fig. S2 at https://drive.google.com/file/d/1co1IRzbSxg8RTYYbUVCuJN1_xxdnX92t/view?usp=sharing). It was hypothesized that I10 and EBV caused GC via upregulation of gankyrin. Ectopic overexpression of gankyrin, while taken 2 μg vector DNA which have shown the stable expression (Fig. 6C). Furthermore, to validate that coinfection of I10 and EBV upregulated the expression of gankyrin which in turn changed the cell microenvironment to promote cell proliferation was performed. Interestingly, we observed the increased number of viable cells at 12, 24, 36, and 48 hpi in infection-II, -III, and -IV in comparison to infection-I and uninfected AGS (Fig. 6D).

Importantly, the ectopic expression of gankyrin not only enhanced the cell proliferation but played a role through interaction with various cellular pathways. It is known to act differentially on various cellular mechanisms such as cell migration, cell cycle regulation, gastric cancer progression, and expression of tumor suppressor genes. Exogenous expression of gankyrin significantly upregulated the cell migratory genes, namely, *mmp3* and *mmp7* (Fig. 6E), and cell cycle regulators like *akt* and *ccnd1* (Fig. 6F and G) showed that the ectopic expression of gankyrin enhanced the expression of *hurp*. Interestingly, the expression of tumor suppressor genes *pten*, *apc*, and *pRB* was significantly downregulated in an exogenous overexpression scenario (Fig. 6H). Furthermore, the exogenous gankyrin increased the expression of GC marker genes *ccl8*, *tff-2*, and *cdx2* while causing a downregulation of *gastrin* (Fig. 6I).

### Knockdown of gankyrin decreased the oncogenic properties of gastric epithelial cells.

**FIG 7:**
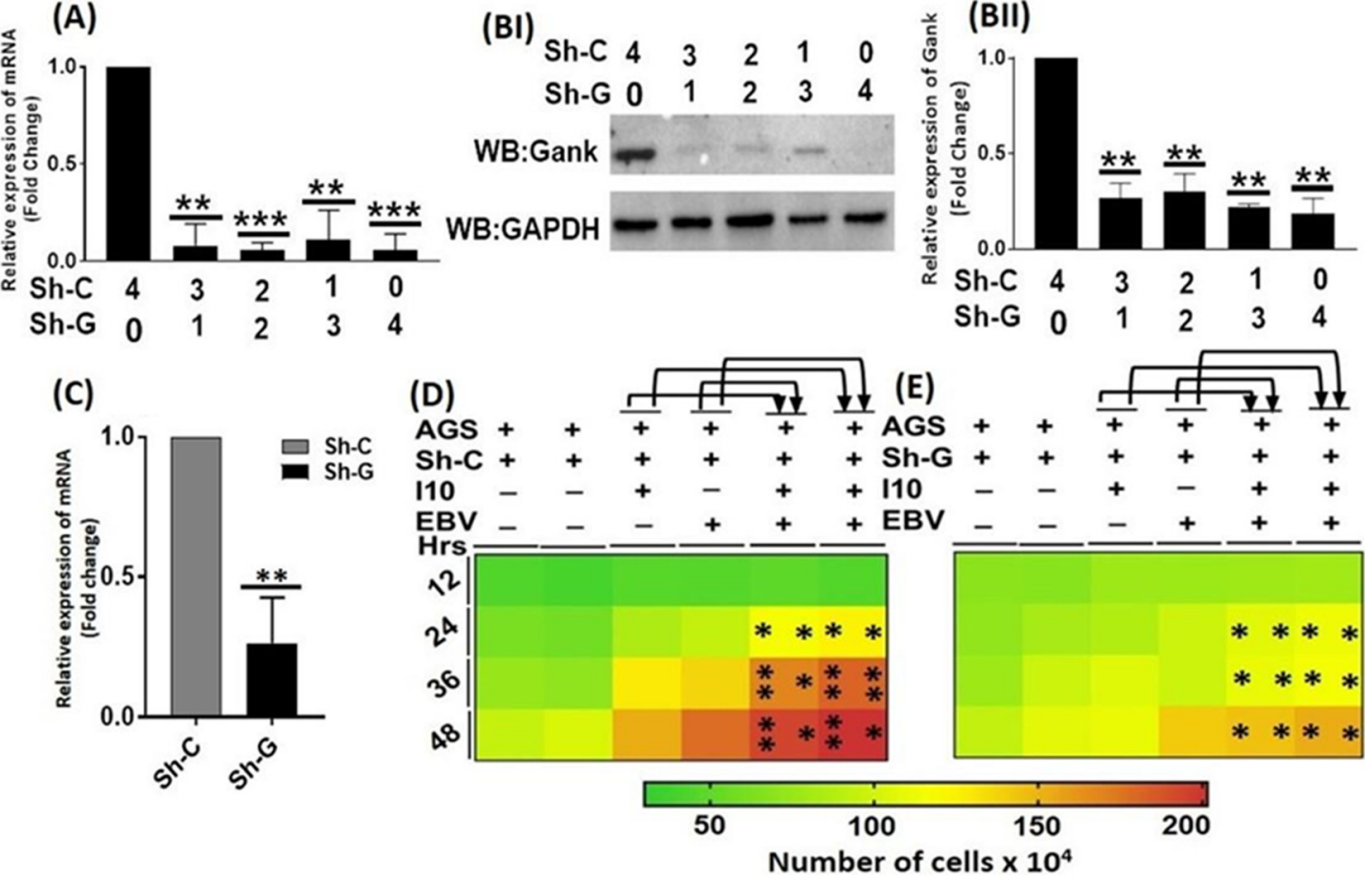
In the presence of H. pylori and EBV, knockdown of gankyrin decreased the oncogenic properties of gastric epithelial cells. (A) Illustration of the relative expression of gankyrin transcripts in a dose-dependent manner. (BI) Representative Western blot image showing the decreased expression of gankyrin in a concentration-dependent manner. (BII) Relative quantification of protein blotting through Image J. (C) Relative transcript expression of gankyrin while given the transfection of 2 μg of sh-G plasmid. (D and E) Interestingly, heat map represents the increased cell proliferation rate in sh-C (D), while upon knockdown of gankyrin, there was a significantly decreased cell proliferation compared to sh-C (E). The experiment was performed for two biological and one technical replicate, and the results are shown as the mean ± SD from two independent experiments.

The study was further extended to understand the role of gankyrin in cellular processes such as cell signaling, cell proliferation, foci formation, and cell migration properties through its knockdown in AGS cells. We optimized the knockdown of gankyrin and observed that 2 μg sh-gankyrin (sh-G) was sufficient for efficient knockdown. qRT-PCR analysis revealed a decreased expression of gankyrin transcripts, after transfection of sh-G at various doses (Fig. 7A). Furthermore, we determined similar results for protein levels (Fig. 7BI, BII, and C). Later, we observed cell proliferation after transfection of sh-G followed by the infection by I10 and EBV (we used above-described infection models) of AGS cells. Interestingly, we noticed an enhanced cell proliferation upon transfection with sh-C followed by I10 and EBV exposure. Also, decreased AGS cell proliferation was observed after the knockdown of gankyrin with I10 and EBV infection (Fig. 7D and E).

**FIG 8:**
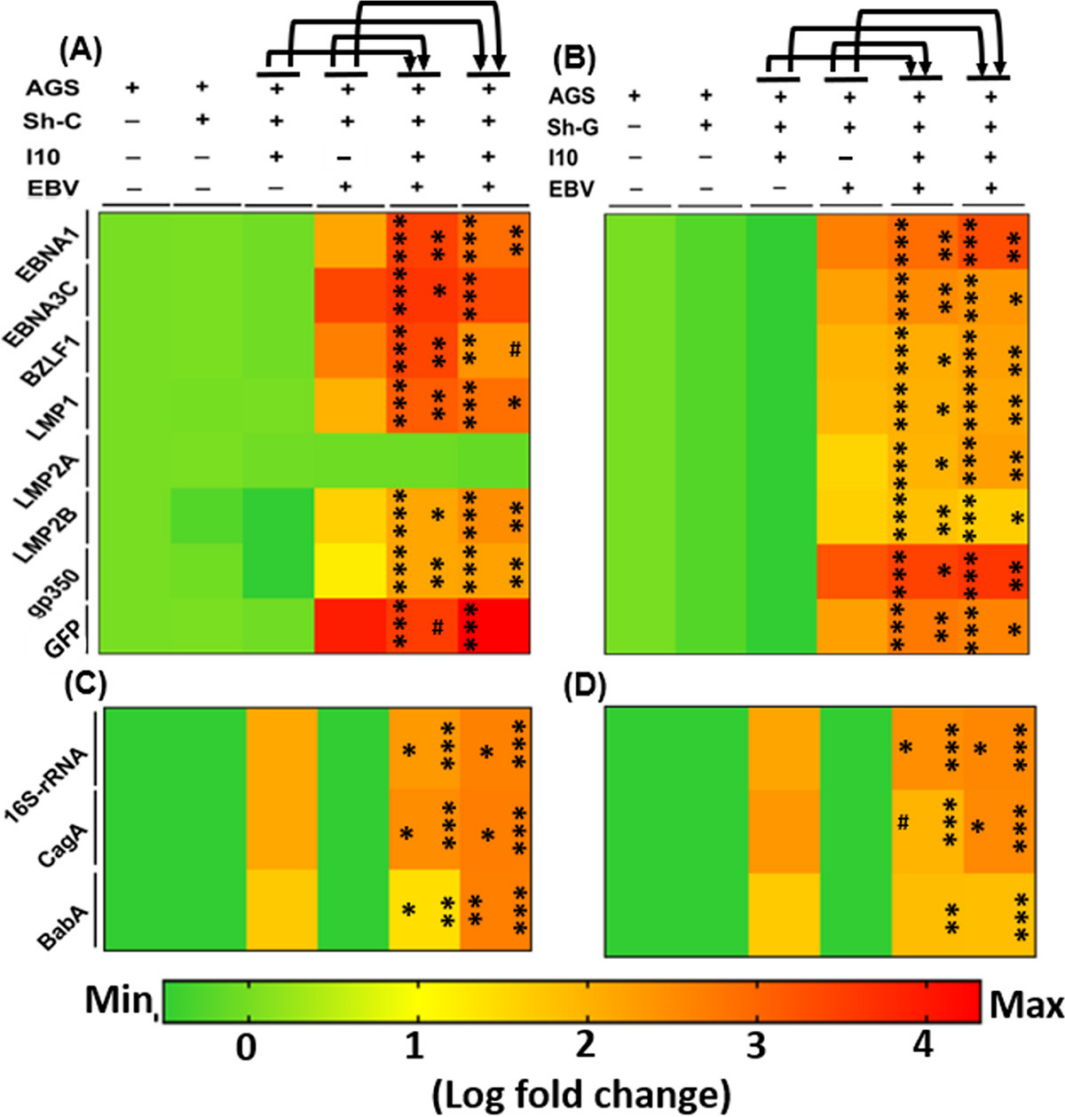
Knockdown of gankyrin modulates the expression profiles of EBV- and H. pylori-associated carcinogenic genes. (A) Heat map shows the relative log fold change expression of EBV-associated latent pathogenic (*ebna1*, *ebna3c*, *lmp1*, *lmp2a*, and *lmp2b*) and lytic (*bzlf1* and *gp350*) gene and EBV-tagged *gfp* gene expression profiles in the presence of sh-C. (B) Heat map represents the qRT-PCR data of EBV-associated latent and lytic and EBV-tagged *gfp* gene expression during knockdown of gankyrin in AGS cells. (C and D) Expression of I10-associated pathogenic gene expression in the presence of sh-C and sh-G, respectively. Importantly, in this experiment we have given the transfection of sh-C or sh-G for 24 h and then provided the infection by I10 and EBV as described in the legend to Fig. 1. The experiment was performed for two biological and two technical replicates, and the results are shown as the mean ± SD for two independent experiments.

Investigation of expression of various latent and lytic genes of EBV and a few important carcinogenic genes of I10 was performed. Abovementioned infection models were studied with an additional panel including sh-C and sh-G. qRT-PCR results showed that the relative expression levels of latent genes *ebna1* and *ebna3c* were higher in infection-III and -IV in comparison to infection-I and -II (Fig. 8A). A significantly increased expression of EBV lytic gene *bzlf1* in infection-III and -IV compared to infection-I was determined. The gene was highly expressed in infection-III and significantly reduced in infection-IV in comparison to infection-II (Fig. 8A). We also determined a significantly elevated expression of latent membrane protein genes *lmp1* and *lmp2b* in infection-III and -IV in comparison to infection-I and -II (Fig. 8A). Additionally, nonsignificant change was observed in *lmp2a* expression (Fig. 8A) and an increased expression of *gp350* in infection-III and -IV was noticed compared to infection-I and -II. Also, an increased expression of *gfp* in infection-III and -IV compared to infection-I was noticed (Fig. 8A). Nonetheless, almost a similar expression pattern of EBV-associated genes with knockdown of gankyrin in AGS cells in infection-III and -IV was determined compared to infection-I and -II (Fig. 8B). The expression of *lmp2a* and *gp350* was higher in the case of sh-G followed by infection, and a decreased expression of *gfp* was obtained compared to sh-C (Fig. 8A and B).

Additionally, we checked the expression profile of I10-associated pathogenic genes, namely, 16S rRNA, *cagA*, and *babA*. qRT-PCR results showed a similar expression of 16S rRNA and *cagA* in infection-III and -IV in comparison to infection-I and -II under both sh-C and sh-G transfection conditions and in abovementioned infection models, unlike in infection-III of sh-G, where we recorded the significantly decreased expression of *cagA* in comparison to infection-I (Fig. 8C and D). Later, we checked the expression of *babA* and noticed that the expression of this gene was higher in infection-III and -IV than in infection-I and -II under the sh-C transfection condition. Meanwhile, in the case of sh-G we observed a similar expression pattern of the *babA* gene in infection-III and -IV (Fig. 8C and D).

**FIG 9:**
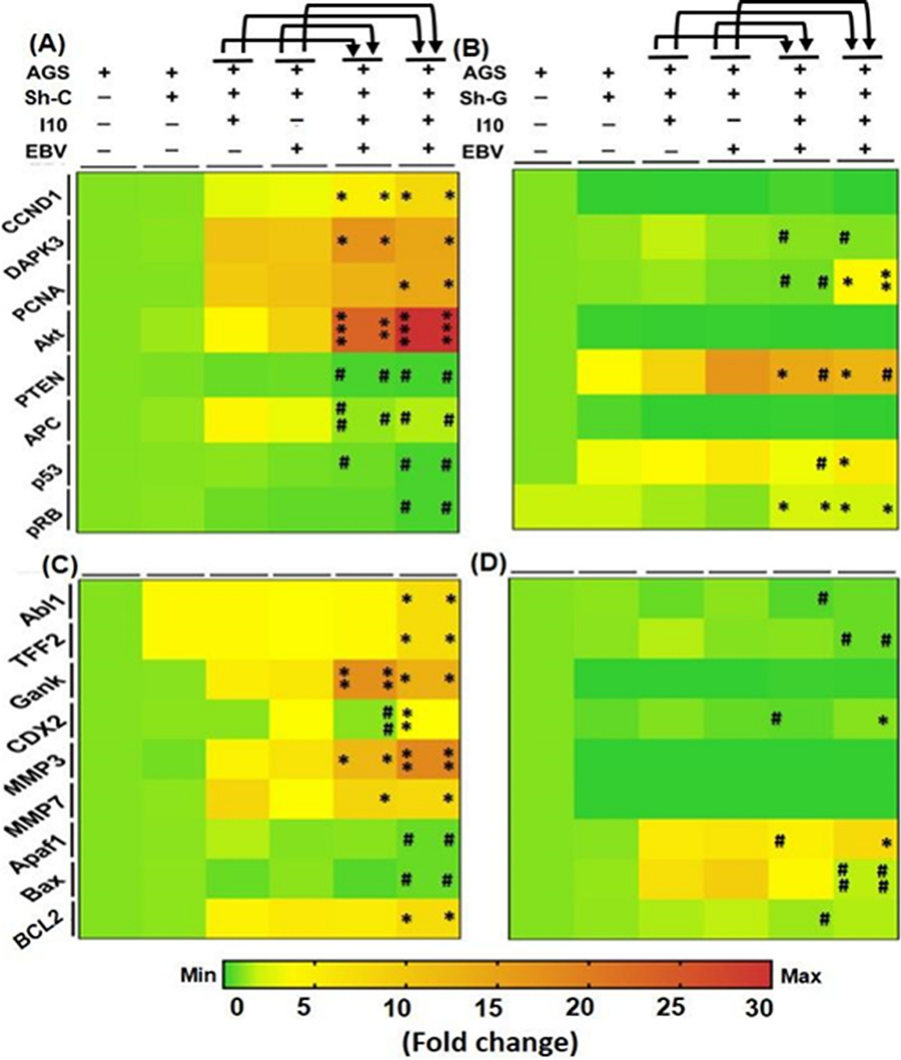
Even in the presence of I10 and EBV, knockdown of gankyrin decreases the oncogenic properties of gastric epithelial cells through the regulation of cell signaling genes. (A) Heat map showing the relative expression of cell cycle regulator (*ccnd1*, *dapk3*, *pcna*, and *akt*) and tumor suppressor (*pten*, *apc*, *p53*, and *pRB*) genes. (B) Expression profiles of abovementioned genes in gankyrin knockdown cells. (C and D) Furthermore, heat map showing the qRT-PCR results of relative transcript expression of gastric cancer (*abl1*, *tff-2*, and *cdx-2*) and DNA damage response (*apaf1*, *bax*, and *bcl2*) genes in all the studied infection models in sh-C vector control and sh-G gankyrin knockdown AGS cells, respectively, after 24 h of infection. The experiment was performed for two biological and two technical replicates, and the results are shown as the mean ± SD for two independent experiments.

Analysis of cell cycle regulators, tumor suppressor, gastric cancer markers, and DNA damage response genes was performed on the knockdown of gankyrin. qRT-PCR results showed a significant increase in expression of cell cycle regulatory genes. Furthermore, we determined a decrease in expression of these genes in infection-I to -IV in gankyrin knockdown cells (Fig. 9A and B). Additionally, we observed increased expression of tumor suppressor genes *pten*, *apc*, *p53*, and *pRB* in sh-G-transfected AGS cells with the abovementioned models (Fig. 9A and B). Later, we determined the expression of GC markers (*abl1*, *akt*, *gank*, and *cdx2*) and cell migratory genes (*mmp3* and *mmp7*) in sh-G-transfected AGS cells. qRT-PCR results showed an increased expression of GC genes *abl1* and *tff-2* in infection-IV in comparison to infection-I and -II (Fig. 9C), whereas the expression of gankyrin was significantly higher in infection-III and -IV than in infection-I and -II (Fig. 9C). Nonetheless, we noticed an increased expression of cell migratory genes *mmp3* and *mmp7* in infection-III and -IV in comparison to infection-I and -II (Fig. 9C). We observed a notable decrease in expression of proapoptotic gene *apaf1* in infection-IV in comparison to infection-I. Also, the *bax* transcripts were reduced in infection-IV compared to infection-II (Fig. 9C), and an increased expression of antiapoptotic gene *bcl2* (Fig. 9C) was recorded. In the case of gankyrin knockdown, we determined a decreased expression of GC marker genes (*abl1*, *akt*, *gank*, and *cdx2*) and cell migratory genes (*mmp3* and *mmp7*) (Fig. 9D). Furthermore, we noticed an elevated expression of proapoptotic gene *apaf1* in infection-IV in comparison to infection-I, while we observed a significantly decreased expression of *bax* in infection-IV compared to infection-I and -II. Furthermore, we determined a decreased expression of antiapoptotic gene *bcl2* in infection-III compared to infection-II (Fig. 9D). Conclusively, on gankyrin knockdown the expression of GC markers and cell migratory and antiapoptotic genes was reduced while the expression of proapoptotic genes remained elevated (Fig. 9C and D).

### EBV and H. pylori coinfection enhanced cell migration through gankyrin.

**FIG 10:**
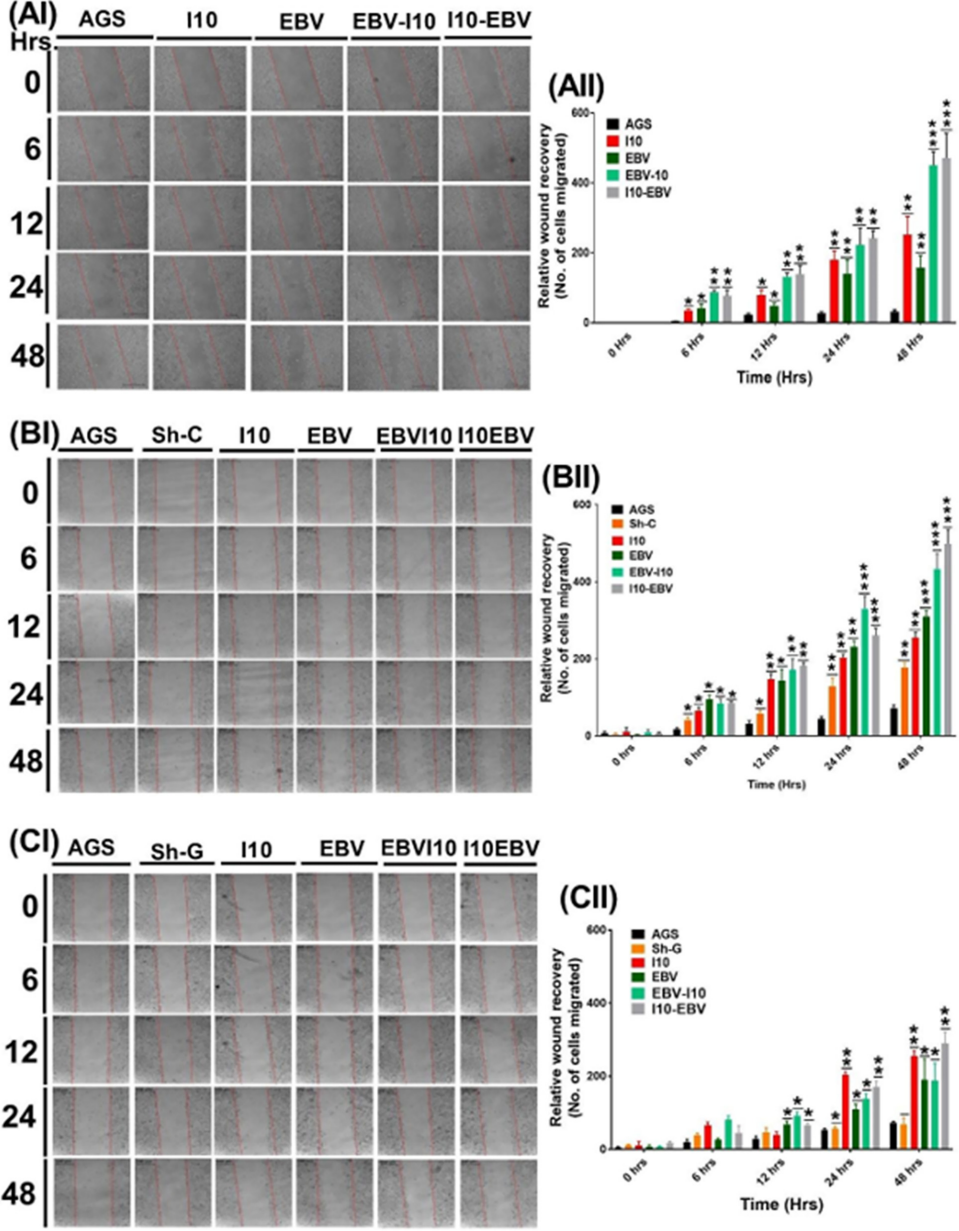
Coinfection by EBV and H. pylori enhanced the cell migratory properties of AGS cells, linked with the expression profiles of gankyrin. (AI) Representative image of scratch wound healing assay. First column shows the uninfected AGS cells. Second, third, fourth, and fifth columns show infection-I, -II, -III, and -IV, respectively, for 0, 6, 12, 24, and 48 h postinfection. (AII) Graphical representation of the number of cells migrated in the wound area. (BI) The image shows the scratch wound in the presence of sh-C vector control followed by infection-I to -IV. (BII) Relative number of cells migrated in the wound area. (CI) Image represents the relatively lower wound recovery in AGS cells during knockdown of gankyrin in AGS cells. (CII) Quantitative graphical representation of the number of cells migrated toward the wound area.

Whether the cellular physiological changes elicited by EBV and H. pylori contributed to cell migration was investigated through cell migration assay. The assay analyzed the migratory properties induced in AGS cells on I10 and EBV infection. We observed cell migration in infection-III and -IV (Fig. 10AI and AII) at all the selected time points. Additionally, we performed the same experiment using knockdown of gankyrin in AGS cells (Fig. 10BI and BII). Importantly, we observed a decreased migration of AGS cells after gankyrin knockdown with the abovementioned models (Fig. 10CI and CII).

### Gankyrin promoted oncogenic properties in AGS cells.

**FIG 11:**
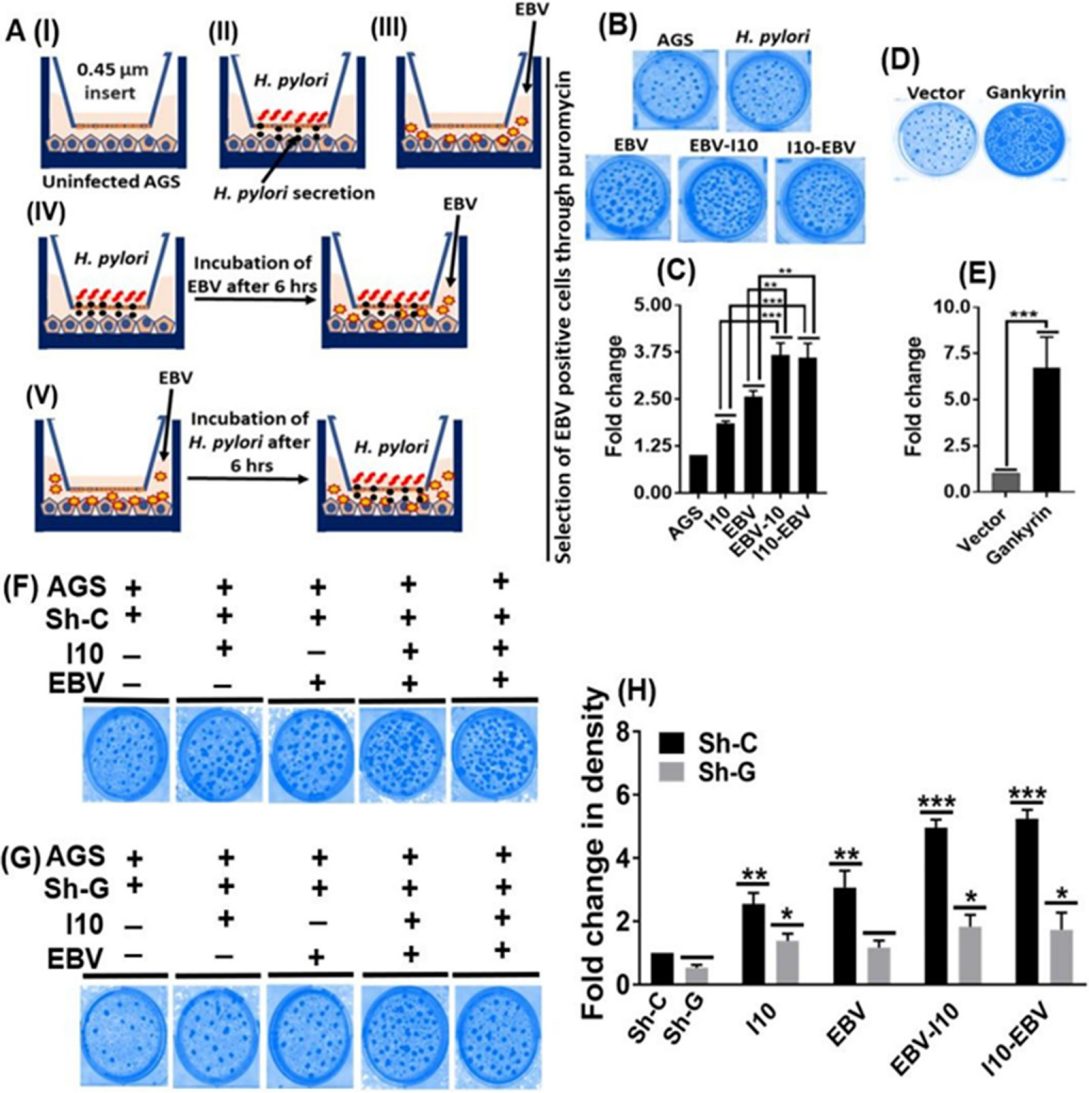
Expression profiles of gankyrin directly linked with tumorous properties of gastric epithelial cells. (A) (I) Uninfected AGS cells. (II) AGS cells with I10 infection. (III) AGS cells with only EBV infection. (IV) First infection by I10 for 6 h followed by second infection by EBV. (V) First infection by EBV for 6 h followed by second infection by I10. Selection of EBV-positive colonies with 2 μg/ml puromycin for 14 days. (B) Representative image of focus formation after following all the infection models. (C) Quantification and graphical representation of density of foci through the Image J software. (D) Further, we have validated focus formation results through the ectopic expression of gankyrin in AGS cells and selected the gankyrin-positive cells through 1 mg/ml Geneticin (G-418) for 14 days. Representative image of foci in exogenously overexpressed AGS cells. (E) Quantification and graphical representation of density of foci in exogenously overexpressed AGS cells through the Image J software. (F and G) Representative image shows the foci in AGS cells after the transfection of sh-C and sh-G, respectively, followed by the infection by I10 and EBV. (H) Moreover, the density of foci in sh-C is higher than in gankyrin knockdown cells. The experiment has been performed two times, and the results are shown as the mean ± SD from two independent experiments.

We performed a clonogenic assay which enabled the selection of EBV-positive cells, further contributing to the understanding of oncogenic transformation efficiency under coinfection conditions. We used a transwell that allowed only the I10 secretory molecules to reach the AGS cells. The experimental models are as depicted in Fig. 11A. A lower number of foci were observed in uninfected and I10-infected AGS cells (Fig. 11B). Moreover, the number of the colonies was comparatively higher in EBV-infected AGS cells and highest under coinfection conditions (Fig. 11B). Further, we quantified the colonies through analysis of colony density and observed that the density of colonies was comparatively high in I10- and EBV-coinfected samples (Fig. 11C). Thus, the secretory molecules produced by I10 may be linked to increased EBV particles in AGS cells. To further validate our findings that the coinfection by I10 and EBV promoted oncogenic properties through the upregulation of gankyrin, exogenous overexpression of gankyrin in AGS cells was done. It was determined that the ectopic expression of gankyrin enhanced the clonogenic properties in AGS cells (Fig. 11D and E). Furthermore, we observed a significantly lower number of foci in gankyrin knockdown AGS cells compared to sh-C (Fig. 11F and G). Density of the colonies was represented in Fig. 11H.

## DISCUSSION

Chronic or acute infection of pathogens that might destroy the immune system is a severe threat to human health and can cause diseases like Alzheimer’s disease, meningitis, cancer, etc. (21–23). Infectious agents not only induce carcinogenesis but may also promote cancer aggressiveness (24, 25). About one-fifth of the total human oncogenesis is associated with infectious agents. Pathogens modulate the cellular milieu by dysregulating the host gene expression, making the environment fit for potential carcinogenesis (26, 27). Studies suggested that I10 and EBV promoted oncogenesis by reframing the expression of cellular genes at transcription and translation levels in infected cells (2). Reprogramming of expression of genes and proteins could promote the intrusiveness of cancer lesions. In this study, AGS cells were used which potentially mimicked the primary gastric epithelial cells. Moreover, it is well established that I10 and EBV infected AGS cells and NCI-N87 cells with equal efficiency (2, 27). Thus, it was intriguing to investigate the synergistic effects of I10-exposed AGS cells which could favor EBV infection. We studied the plausible effects of the EBV-infected AGS cell microenvironment that could favor I10 infection.

It is reported that I10-exposed cellular environmental conditions were suitable for an enhanced EBV infection and vice versa (2, 27). In studies by Pandey et al. and Sonkar et al., it was reported that these pathogens dysregulated the cellular genes’ transcript expression which led to aggressive GC (2, 27). Moreover, to date it is an enigma whether the prior exposures to I10 created a suitable niche for the EBV infection or whether an anterior windage of EBV created a niche favorable to aggressive growth of I10 leading to GC. Notably, Pandey et al. also reported an increased expression of green fluorescent protein (GFP) in the presence of CagA-positive H. pylori (2). In our study we found that I10 and EBV coinfection enhanced the expression of pathogenic genes such as *ebna1*, *ebna3c*, *bzlf1*, *gp350*, *lmp1*, *lmp2a*, *lmp2b*, and *gfp* at various time intervals. Furthermore, we recorded an increased expression of *gfp* in schemes III and IV which correlated with elevated EBV copy numbers. We determined that the expression of EBV-associated latent genes can promote the replication and spread of the virus under coinfection conditions up to 48 h, unlike *ebna3c*. Various studies reported that *ebna1* and *ebna3c* enhanced the G_1_/S-phase transition, cell invasiveness, and metastasis through the upregulation of cell signaling genes such as *Slug*, *Snail*, *Vimentin*, and *E-cadherin* (28–31). Additionally, it is also known to regulate tumor suppressor genes like *p53* and *pRb*. The gene expression pattern in I10 and EBV coinfections revealed that I10 contributed to an increased replication and transcription of EBV pathogenic genes and virion production. Notably, we observed that expression of another latent gene, *ebna3c*, was significantly correlated with the expression of oncogenic protein gankyrin at 12, 24, and 48 hpi. We performed Western blot analysis in which we observed a similar expression pattern of gankyrin as that in qRT-PCR. These results revealed that *ebna3c* may be directly linked with the expression of gankyrin as the expression of *ebna3c* and gankyrin was alleviated only in the presence of EBV. These results supported that *ebna3c* may cause GC through the regulation of gankyrin (15, 16). It is noteworthy to state that there is an absence of reports which support that an infectious agent caused GC through upregulation of gankyrin. However, there is need of further research to validate a direct interaction between gankyrin and *ebna3c*. A study had reported that gankyrin is generally expressed due to oxidative and cellular stress environments created by different factors including the pathogens at earlier time points. Furthermore, after the coinfection by I10 and EBV we observed an intact cellular morphology up to 48 hpi; however, later cellular morphology was distorted successively due to the heavy load of these two pathogens and unavailability of nutrients. Hence, the continuation of the experiment beyond 48 h was considered inappropriate for the experiments.

This is the first study which demonstrated that the exposure of gastric epithelial cells to EBV before I10 and vice versa increased the expression of gankyrin, leading to aggressive gastric carcinogenesis. 16S rRNA indicated establishment of I10 infection in AGS cells. In the present study, we determined that the human gastric epithelial cells have enhanced susceptibility to EBV infection in the presence of the *cagA*-positive I10 strain. The I10-associated *cagA* secretory antigen encoded by the *cagA* pathogenicity island (*cagA-*PAI) is present in about 89% of H. pylori strains and is known to cause morphological dysplastic alteration in epithelial cells (32, 33). Dysplastic changes contribute to the initiation of carcinogenesis. Furthermore, I10 carrying *cagA* is associated with inflammation, peptic ulcers, gastritis, and GC (26). Pandey et al. showed that a *cagA* mutant strain of H. pylori had a significant reduction in EBV titer production (2). This study suggested that instead of the *cagA* mutant, wild-type I10 could contribute significantly to viral replication and subsequently cancer progression and aggressiveness. I10 is loaded with a type IV bacterial secretion system that helps in the injection of its associated secretory protein into the host cells. An alternative mode of uptake of *cagA* is through integrin-mediated rearrangements of actin filaments and endocytosis. Endocytosis of *cagA* changed the intracellular milieu and led to morphological alterations in the gastric epithelial cells. Here, our results supported the hypothesis that the type IV secretory mechanism was used by this bacterium, as the direct incubation with wild-type I10 showed enhanced infectivity. The expression of 16S rRNA and *cagA* was higher in coinfected samples, which showed that EBV could also help synergistically to increase the expression of I10-associated factors. Expression of 16S rRNA showed that enhanced replication and multiplication of the I10 genome were possible in the presence of EBV. Furthermore, *babA* transcripts, known to be highly expressed in peptic ulcer and GC rather than asymptomatic colonization, were highly expressed in infection-I. Further, a higher *babA* expression at early time points (12 and 24 hpi) suggested an increased infectivity of I10 as it helped in the attachment of I10 with host cells.

In addition to the above results, the recruitment of gankyrin upon coinfection altered the downstream cell signaling genes which are known to be directly influenced by overexpression of this protein. These genes were studied under six categories: cell cycle regulator, GC marker, cell migratory, tumor suppressor, DNA damage response, and antiapoptotic genes. Besides the ectopic expression of gankyrin, knockdown of this gene decreased the cancerous properties of gastric epithelial cells. Furthermore, we found that *ebna3c* and the gankyrin gene could modulate the status of various cellular proteins which played a significant role in GC. However, experiments need to be conducted to validate a direct relation of *ebna3c* and gankyrin. Additionally, cell cycle regulatory genes are the hallmark of cancer progression and metastasis. Results of the present study suggested that differential expression of cell cycle regulatory genes such as *ccnd1*, *dapk3*, *pcna1*, and *akt* in all the infection models at various time points could be linked to increased oncogenesis. Gankyrin is known to upregulate *ccnd1* and *pcna* in pancreatic cancer and GC, respectively (34). Besides these genes, the expression of gankyrin upregulated the *akt* gene and protein in hepatocellular cancer, liposarcoma, and GC (35–37). The upregulation of *akt* through the action of gankyrin enhanced cell proliferation, metastasis, stem cell self-renewal capacity, autophagy, and chemoresistance (18, 38). The upregulation of *dapk3* is linked with enhanced apoptosis. Coinfection by these two oncogenic pathogens downregulated *dapk3*, which disrupted the homeostasis in cell proliferation and apoptosis, consequently leading to increased cell number. Results showed a significantly lower expression of gankyrin at 12 and 24 hpi than at 48 h. Furthermore, this finding supported that I10 and EBV infection mediated higher expression of gankyrin which led to aggressive GC through the dysregulation of cell cycle regulatory genes. Importantly, shRNA-mediated knockdown of gankyrin decreased the expression of cell cycle regulatory genes and further alleviated the proliferation of AGS cells.

I10 and EBV can hijack nonreceptor tyrosine kinase (Abl1) signaling to remodel the host cytoskeleton for multiple purposes such as late-phase autophagy, cell motility, and cell adhesion. *tff-2* is known to be overexpressed in GC. It is a structural component of gastric mucosa and inhibits gut acid secretion and its motility. Inhibition of acid secretion can provide a suitable platform for rapid growth of I10, which could further contribute to EBV reactivation and replication (39, 40). CDX-2 preferentially binds to methylated DNA and is involved in multiple cellular processes such as transcriptional regulation of genes of epithelial cells. In the present study, analysis of GC marker genes suggested that I10 changed the status of these genes for its growth and development of oncogenesis. It not only was involved in the dysregulation of host oncogenic factors but also changed the expression of genes that facilitated a suitable microenvironment for its growth and replication. The overexpression of *mmp3* and *mmp7* in infection models indicated a probable role of I10 and EBV in gankyrin-mediated aggressiveness of GC through the upregulation of such kinds of cell migratory genes. Notably, matrix metalloproteinases (MMPs) are hallmarks of cancer progression and metastasis; meanwhile, the knockdown of gankyrin in AGS cells decreased wound healing properties which are potentially linked with an elevated growth and transformation of gastric epithelial cells. Hence, gankyrin could be an important biomarker whose expression status is potentially linked to oncogenesis.

Tumor suppressor genes comprise the defense system of the body that helps control abnormal growth and proliferation of cells through various mechanisms including the promotion of DNA repair, apoptosis, or modulation of cell signaling. Downregulated *pten* is associated with several types of cancers and functions as a negative regulator of the phosphatidylinositol 3-kinase (PI3-kinase)/Akt pathway by downregulating PIP3 (phosphatidylinositol 3,4,5-triphosphate). Gankyrin downregulated the tumor suppressor protein-16 (*p16*), protein 53 (*p53*), and protein retinoblastoma (*pRB*) genes through direct interaction with various domains (17). The loss of activity of these tumor suppressor genes is crucial for carcinogenesis. This demonstrated an association of I10 and EBV infection in gankyrin upregulation, the cellular genome, and dysregulation of cellular signaling leading to oncogenesis. Besides the overexpression of this gene, knockdown of gankyrin restored the expression of tumor suppressor genes. Moreover, upon the knockdown of gankyrin we observed an alleviated focus formation. *Apaf1* and apoptosis regulator (*bax*) profiles after coinfection suggested that the coinfection by I10 and EBV downregulated the proapoptotic genes via upregulating gankyrin. Furthermore, I10 and EBV infection mediated upregulation of gankyrin to further downregulate the expression of antiapoptotic *bcl2*. Notably, we recorded the decreased expression of antiapoptotic gene *bcl2* and elevated expression of proapoptotic genes in AGS cells during the knockdown of gankyrin.

### Conclusion.

**FIG 12:**
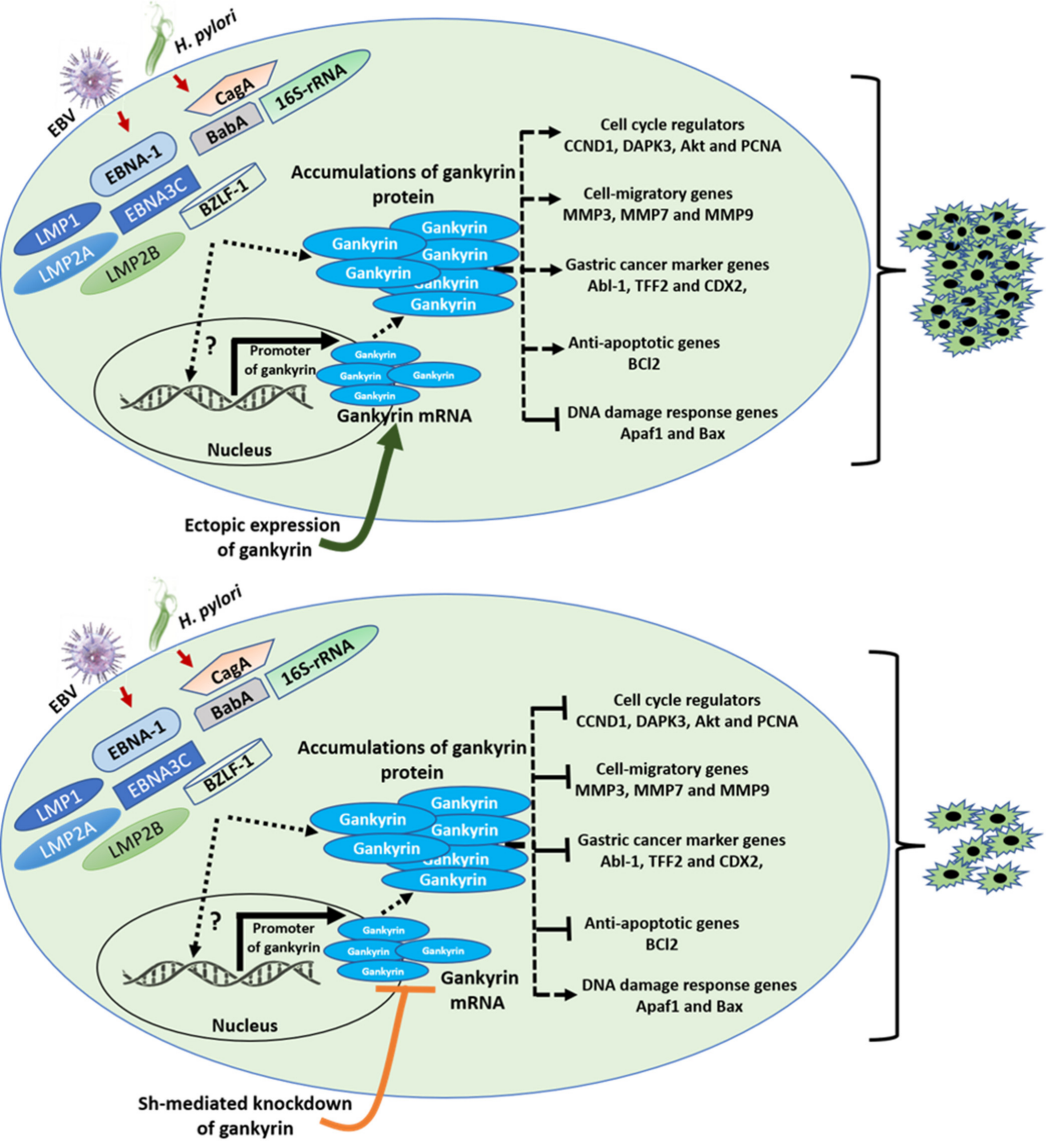
A model illustrating the association of gankyrin in I10- and EBV-mediated gastric cancer. This association drives the progression of I10- and EBV-mediated gastric cell transformation. Oncogenic activity of gankyrin is accelerated in the presence of pathogens which target the cell cycle regulators, cell migratory genes, gastric cancer markers, antiapoptotic genes, DNA damage response genes, and tumor suppressor genes. Besides coinfection-mediated and ectopic expression of gankyrin in AGS cells, knockdown of gankyrin in AGS cells decreased the oncogenic properties of gastric epithelial cells. Hence, gankyrin could be a potential oncogenic protein which may potentially be involved in the aggressiveness of gastric cancer mediated by I10 and EBV.

The current study demonstrated that I10- and EBV-associated factors specifically enhanced the expression of gankyrin. Upregulated gankyrin mediated dysregulated expression of cell cycle regulator, GC marker, cell migration, DNA response, and antiapoptotic genes in infected gastric epithelial cells. I10 and EBV created an intracellular and extracellular milieu which potentially favored gastric epithelial cell proliferation and carcinogenesis. Together, I10 and EBV contributed to a suitable tumor microenvironment represented by elevated colony formation and wound recovery assay. Additionally, gankyrin might be playing a central role in the transformation process through altered regulation of various host genes (Fig. 12). Meanwhile, more study needs to be conducted to check the direct interaction of gankyrin with EBV-associated factors.

## MATERIALS AND METHODS

### Cell culture.

The gastric cancer EBV-negative cell line (AGS) was obtained from the National Center for Cell Science (NCCS), Pune, India. The cells were cultured in Dulbecco’s modified Eagle’s medium (DMEM; Himedia, Mumbai, India) containing 10% fetal bovine serum (FBS; BioWest, South America origin) and 100 U/ml penicillin-streptomycin (Himedia, Mumbai, India) under specific conditions of 5% CO_2_ and humidified air at 37°C (Eppendorf India).

### EBV culture and isolation.

Human embryonic kidney (HEK293T) cells containing GFP-EBVWT (obtained from Erle S. Robertson’s laboratory, University of Pennsylvania, USA) were cultured and used to obtain EBV (2, 25). Concisely, HEK293T cells containing the GFP-EBVWT were selected using puromycin and further induction was given to HEK293T EBV cells for 4 to 5 days with 3 mM butyric acid and 20 ng/ml tetradecanoyl phorbol acetate (TPA) (Sigma-Aldrich Corp., St. Louis, MO). Concentrated virus was obtained through the ultracentrifugation of HEK293T EBV cell culture supernatant at 23,500 rpm and 4°C for 90 min (41). The multiplicity of infection (MOI) was determined by infecting the AGS cells at 40 to 45% confluence in a 6-well plate with virus amounts (25, 50, 75, 100, 125, and 150 μl) corresponding to MOIs of 2.5, 3, 3.5, 4, 4.5, and 5. Further, we calculated and used a MOI of 2.5 for the infection of 40 to 45% confluent AGS cells in a 6-well plate as well as validation through qRT-PCR and detection of *gfp* and *ebna1* (27, 42).

### Bacterial (H. pylori) culture.

The I10 strain (I10) was a kind gift from Asish Kumar Mukhopadhyay (National Institute of Cholera and Enteric Diseases, ICMR, Kolkata, India). Single colonies from the I10 culture plates were mixed in 3 ml brain heart infusion medium (catalog no. 237500; BD Difco brain heart infusion broth), containing 10% FBS (Himedia; catalog no. RM 10432) and 1× I10 selective antibiotics (10 mg/liter vancomycin, 5 mg/liter amphotericin B, 5 mg/liter cefsulodin, and 5 mg/liter trimethoprim) in a snap-cap tube (BD; catalog no. 352001). Culture tubes were kept in a microaerobic workstation and incubator (Whitley DG250) to provide microaerophilic conditions (85% N_2_, 10% CO_2_, and 5% O_2_) at 37°C.

### Coinfection of EBV and H. pylori with AGS cells.

For infection, we took 0.25 × 10^6^ AGS cells and cultured them in a 6-well plate. Further, we observed the cells up to 45 to 50% confluence and then incubated them with a MOI of 100 of I10 and 25 μl of EBV corresponding to a MOI of 2.5. For this study, we developed five independent approaches for the construction of coinfection models using I10 and EBV. A detailed description is given in the introduction. We collected all the infected samples after 12, 24, and 48 hpi. Furthermore, we used two additional schemes, sh-C and sh-G, with the abovementioned models. In this experiment, the uninfected AGS cells were taken as negative control. The uninfected cells confirmed the absence of contamination of the two pathogens, and thus, the mock was uncompromised.

### RNA isolation and qRT-PCR.

Following all four abovementioned infection models, fixed numbers of I10 (MOI of 100) and EBV (MOI of 2.5) were incubated with AGS cells under specific cell culture conditions (5% CO_2_, 37°C) for 12, 24, and 48 h. Further, total RNA was isolated, and qRT-PCR was performed as described earlier (27, 42). Subsequently, pathogenic and host genes were analyzed.

### Lentiviral shRNA-mediated gene silencing.

The sense strand of gankyrin shRNA sequences was 5′-tcgagtgctgttgacagtgagcgaGCTGTACTCCCTTACATTATGtagtgaagccacagatgtaCATAATGTAAGGGAGTACAGCgtgcctactgcctcggaa-3′. Here, the sequence in uppercase designates the gankyrin target sequence and the lowercase letters denote the hairpin along with the sequences which are essential for the directional cloning in pGIPZ vector. These single-stranded oligonucleotides were individually cloned into the pGIPZ vector using XhoI and MluI restriction sites. Additionally, a control shRNA sequence, 5′-TCTCGCTTGGGCGAGAGTAAG-3′, was used to make sh-Ctrl vector which lacked the complementary sequences in the human genome (42, 43). Meanwhile, pGIPZ vector was puromycin resistant and GFP tagged. Thus, GFP immunofluorescence was observed through an Olympus 1X83 microscope using a 560-nm excitation and 645-nm emission filter. Puromycin-selected cells were grown up to 90% confluence, and the expression levels of target proteins were checked.

### Western blotting.

Western blot experiments were carried out as described earlier (42, 43). The lysates were analyzed using an antigankyrin polyclonal primary antibody (cell signaling) and horseradish peroxidase (HRP)-tagged secondary antibody. Blots were observed under a ChemiDoc XRS+ system with Image Lab software no. 1708265.

### Immunofluorescence assay.

An immunofluorescence assay was performed as described earlier (44) by using an antigankyrin primary antibody, and specific signals were detected with secondary antibodies conjugated with Alexa Fluor 488 (cell signaling). The cell nucleus was counterstained with 4′,6′-diamidino-2-phenylindole (DAPI). The images were obtained using confocal microscopy (Olympus IX83) at 60×, zoomed twice for convenience of analysis.

### Cell proliferation assay.

The cell proliferation assay was accomplished through trypan blue exclusion methods as described earlier. Briefly, 25 × 10^4^ AGS cells were infected with I10 (MOI of 100) and EBV (MOI of 2.5) as described in infection models incubated at 37°C for 12, 24, 36, and 48 h, while the uninfected AGS cells were taken as control. After the abovementioned infection time, the cells were trypsinized and mixed with trypan blue (1:1). Furthermore, the cells were counted using a hemocytometer. Only viable white cells were counted, and blue nonviable cells were excluded (25).

### Colony formation assay.

AGS cells were infected with I10 and EBV, and EBV-positive cells were selected using puromycin (2 μg/ml). We selected the ectopically expressed gankyrin-positive AGS cells with Geneticin (G-418) at 1 mg/ml, while the sh-gankyrin pGIPZ-positive cells were selected with puromycin. Images were taken on a ChemiDoc XRS+ system with Image Lab software no. 1708265 (40).

### Statistical analysis.

Data were statistically analyzed using a two-tailed Student *t* test. All the results were derived from a set of triplicate experiments. *P* values were estimated using GraphPad Prism version 6, and *P* values of <0.05, <0.01, and <0.001 were considered statistically significant and represented by *, **, and ***, respectively.
